# Triplet Therapy (Androgen Deprivation Therapy + Darolutamide + Biweekly Docetaxel) for High-risk Metastatic Castration-sensitive Prostate Cancer (TRIC Study): A Study Protocol for Open-labelled Single-Arm Phase II Clinical Trial

**DOI:** 10.31662/jmaj.2026-0045

**Published:** 2026-05-08

**Authors:** Hiroaki Iwamoto, Tomohiro Hori, Takahiro Inaba, Ryunosuke Nakagawa, Taiki Kamijima, Hiroshi Kano, Tomoyuki Makino, Renato Naito, Hiroshi Yaegashi, Takahiro Nohara, Kazuyoshi Shigehara, Kouji Izumi, Atsushi Mizokami

**Affiliations:** 1 Department of Integrative Cancer Therapy and Urology, Kanazawa University Graduate School of Medical Science, Kanazawa, Japan

**Keywords:** metastatic castration-sensitive prostate cancer, triplet therapy, darolutamide, docetaxel, androgen deprivation therapy

## Abstract

**Background:**

Triplet therapy consisting of androgen deprivation therapy (ADT), docetaxel, and an androgen receptor signaling inhibitor (ARSI) has demonstrated survival benefits in patients with metastatic castration-sensitive prostate cancer (mCSPC), particularly those with high-risk disease. However, the feasibility of this intensive regimen is often limited by docetaxel-associated toxicity, especially in Japanese patients. Under the current Japanese health insurance system, darolutamide is the only ARSI approved for use in triplet therapy for mCSPC. Therefore, optimization of docetaxel administration is a key clinical issue.

**Methods:**

The triplet therapy (androgen deprivation therapy + darolutamide + biweekly docetaxel) for high-risk metastatic castration-sensitive prostate cancer (TRIC) study is an investigator-initiated, single-center, open-label, single-arm phase II clinical trial designed to evaluate the efficacy and safety of triplet therapy consisting of ADT, darolutamide, and biweekly low-dose docetaxel (35 mg/m^2^ every two weeks) in patients with high-risk mCSPC. High-risk disease is defined according to the castration-sensitive PC classification proposed by Kanazawa University (Canazawa) risk classification, which includes Gleason pattern 5, bone scan index ≥1.5, and lactate dehydrogenase ≥300 IU/L. The primary endpoint is prostate-specific antigen (PSA) response at three and six months. Secondary endpoints include overall survival, time to castration resistance, radiographic progression-free survival, safety, and other clinically relevant outcomes.

**Results:**

This study will prospectively assess the feasibility, safety, and early efficacy of the proposed triplet regimen. PSA response rates, survival outcomes, and treatment-related adverse events will be analyzed to generate preliminary evidence regarding treatment activity and tolerability in this high-risk population.

**Conclusions:**

The TRIC study will provide important prospective data on a modified triplet therapy tailored to the Japanese clinical setting. The findings are expected to inform future randomized trials and to contribute to the development of optimized treatment strategies for patients with high-risk mCSPC.

**Trial registration:**

Japan Registry of Clinical Trials (jRCTs) 041240151, registered: December 18, 2025.

## Introduction

Prostate cancer (PC) is the most frequently diagnosed cancer among men and a leading cause of cancer-related mortality in developed countries ^(1)^. Approximately 10% of patients are diagnosed with de novo metastatic disease at the time of initial diagnosis, classified as metastatic castration-sensitive PC (mCSPC) ^(2)^. Since the introduction of androgen deprivation therapy (ADT) targeting androgen receptor (AR) signaling in 1941, ADT has remained the cornerstone of treatment for metastatic PC ^(3)^. Combined androgen blockade (CAB), which includes a nonsteroidal anti-androgen in conjunction with a luteinizing hormone-releasing hormone (LHRH) agonist or bilateral orchiectomy, was developed to enhance treatment outcomes. In recent years, novel AR signaling inhibitors (ARSIs) have emerged as the standard of care in the management of mCSPC. Current clinical guidelines recommend combination therapy with ADT and ARSIs (e.g., abiraterone, enzalutamide, or apalutamide), or a triplet regimen consisting of ADT, docetaxel (DTX), and an ARSI [e.g., abiraterone or darolutamide (DAR)] for patients with mCSPC ^(4)^. Among these, triplet therapy is considered the most potent treatment option and is strongly recommended (category 1), particularly for patients with high-volume or high-risk disease. At our institution, triplet therapy is implemented for patients classified as high-risk according to our proprietary castration-sensitive PC classification proposed by Kanazawa University (Canazawa) risk classification ^(5), (6)^. In the Canazawa risk classification, patients with 0 or 1 of the following three factors are defined as low-risk patients, while those with 2 or 3 factors are defined as high-risk patients: Gleason pattern 5, bone scan index (BSI) of 1.5 or higher, and lactate dehydrogenase (LDH) of 300 IU/L or higher ^(5), (6)^. However, the feasibility of triplet therapy is often limited by the toxicity associated with DTX. In particular, Japanese patients have been reported to experience a high incidence of severe hematologic adverse events following standard-dose DTX administration ^(7)^. To address this issue, we previously evaluated a biweekly low-dose DTX regimen (35 mg/m^2^ every two weeks) in patients with metastatic castration-resistant PC (mCRPC) and demonstrated acceptable efficacy with improved tolerability compared with the conventional 3-weekly regimen ^(8)^. Based on these findings, this modified regimen has been adopted in our institution as a feasible alternative to standard-dose DTX. On the basis of this clinical background, we hypothesized that a triplet regimen consisting of ADT, DAR, and biweekly low-dose DTX could provide an effective and tolerable treatment option for Japanese patients with high-risk mCSPC. The TRIC study is an open-label, single-arm phase II clinical trial designed to evaluate the efficacy and safety of this combination. The primary endpoint of the study is prostate-specific antigen (PSA) response at three and six months.

## Materials and Methods

### Aim of the study

The aim of this study is to evaluate the efficacy and safety of the triplet therapy consisting of ADT, DAR, and biweekly DTX in patients with high-risk mCSPC.

### Study design

This study is a phase II, investigator-initiated, single-center, open-label, single-arm clinical trial designed to evaluate the clinical utility of the triplet therapy consisting of ADT, DAR, and biweekly DTX in patients with high-risk mCSPC.

### Endpoints

The primary endpoint is PSA response rate, defined as a PSA decline of 95% or more from baseline. Secondary endpoints include overall survival (OS), time to castration resistance (TTCR), radiographic progression-free survival (rPFS), progression-free survival (PFS), undetectable PSA rate, time to neuroendocrine PC (NEPC), time to double negative PC (DNPC), symptomatic skeletal event (SSE)-free survival, time to first SSE, time to pain progression, time to subsequent antineoplastic therapy, time to cabazitaxel, time to performance status progression, time to opioid use ≥7 days, safety, adverse events, and the relationship between chemokine (CC motif) ligand 2 (CCL2) and chemokine (CC motif) ligand 5 (CCL5) concentrations and treatment efficacy. NEPC will be defined based on pathological confirmation when available or on clinical features suggestive of treatment-emergent neuroendocrine differentiation, such as aggressive disease progression with low PSA levels relative to tumor burden or the presence of visceral metastases. DNPC will be defined as treatment-emergent PC showing clinical progression with features of androgen-indifferent disease without evidence of neuroendocrine differentiation ^(9)^. OS is defined as the time from the start of treatment to death from any cause. Surviving patients were censored at the date of last confirmed survival. For patients lost to follow-up, the last confirmed day of survival before loss to follow-up will be the censoring date. TTCR is defined as the time from the start of treatment to the development of castration-resistant PC. rPFS is defined as the time from the start of treatment to radiographic disease progression or death from any cause. Disease progression is based on the Response Evaluation Criteria in Solid Tumors v.1.1 criteria and the Prostate Cancer Working Group 2 (PCWG2) criteria.

### Eligibility criteria: Inclusion criteria

Patients meeting all the following criteria are eligible to participate in this study:

1. Diagnosis of mCSPC.

2. Histologically confirmed adenocarcinoma of the prostate.

3. Age ≥20 years at the time of enrollment.

4. Eastern Cooperative Oncology Group performance status of 0-2.

5. Fulfillment of at least two of the following high-risk criteria:

・Presence of Gleason pattern 5

・BSI ≥1.5

・LDH ≥300 IU/L

6. No active concomitant infectious disease.

7. No fever suspected to be related to infection.

8. No history or evidence of interstitial lung disease or pulmonary fibrosis.

9. Adequate hematologic function, defined as:

・Absolute neutrophil count ≥2,000/mm^3^

・White blood cell count ≥3,000/mm^3^

・Platelet count ≥100,000/mm^3^

・Hemoglobin level >8.0 g/dL

10. Adequate hepatic and renal function, defined as:

・Total bilirubin < upper limit of normal (ULN)

・Aspartate aminotransferase <2.5 × ULN

・Alanine aminotransferase ≤2.5 × ULN

・Serum creatinine <1.5 × ULN

11. Written informed consent obtained from the patient prior to participation, based on the patient’s free will after receiving sufficient explanation of the study.

### Ineligibility criteria: Exclusion criteria

Patients meeting any of the following criteria will be excluded from participation in this study:

1. Prior treatment for mCSPC; however, patients who have received ADT or CAB for ≤3 months before study entry are eligible.

2. Participation in another clinical study or investigational trial within the three months prior to initiation of the study treatment.

3. Known hypersensitivity to ADT, DAR, or DTX.

4. Presence of severe bone marrow suppression.

5. Active infection or suspected infection at the time of enrollment.

6. Severe hepatic impairment classified as Child-Pugh class C.

7. End-stage renal disease requiring dialysis or an estimated glomerular filtration rate (eGFR) <15 mL/min/1.73 m^2^.

8. Any condition that, in the opinion of the principal investigator or sub-investigators, makes the patient unsuitable for participation in this study.

### Treatment

**Figure 1.**
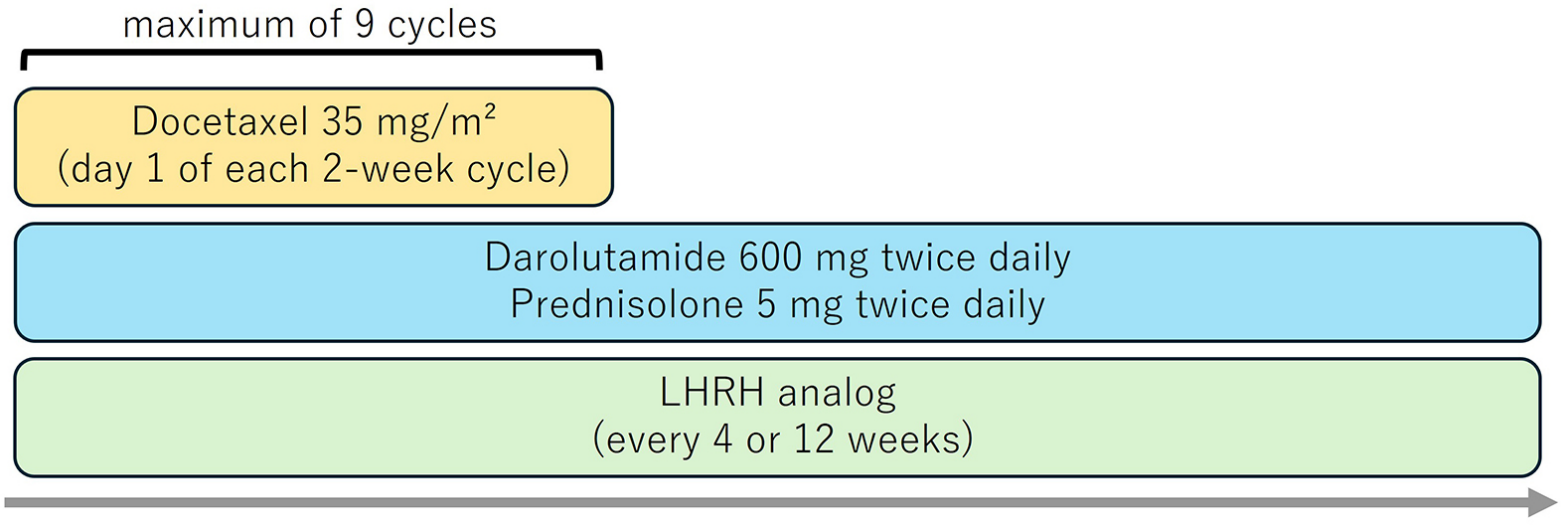
Administration schedule. LHRH: luteinizing hormone-releasing hormone.

In this study, patients receive dexamethasone sodium phosphate (6.6 mg) as a 15-minute intravenous infusion followed by DTX (35 mg/m^2^) as a 60-minute intravenous infusion on day 1 of each 2-week cycle, for a maximum of nine cycles. Prednisolone is administered orally at a dose of 5 mg twice daily. DAR is administered orally at a dose of 600 mg twice daily. ADT with an LHRH analog is continued, with regular administration every four or 12 weeks (Figure 1). After completion of a maximum of nine cycles of DTX, treatment with the oral DAR and LHRH analog is continued. There are no restrictions on subsequent therapies after discontinuation of the study treatment, and standard treatments are administered at the discretion of the attending physician. The use of bone-modifying agents, including zoledronic acid or denosumab, for the management of bone metastases is also determined by the attending physician. As supportive care, granulocyte colony-stimulating factor is initiated in cases of febrile episodes (generally ≥38°C), absolute neutrophil counts <500/mm^3^, or neutrophil counts <1,000/mm^3^, as clinically indicated. Blood transfusions are administered at the discretion of the attending physician in cases of bone marrow suppression. If grade 3 or higher adverse events [Common Terminology Criteria for Adverse Events (CTCAE) version 5.0)] occur during DTX treatment, administration will be withheld until recovery and then resumed at a reduced dose (initially 70% of the original dose). Further dose reductions or treatment discontinuation will be performed if treatment-related toxicities persist or recur and continued administration is considered infeasible.

### Follow-up

Physical examinations, laboratory tests, and adverse event assessments will be performed according to the CTCAE version 5.0 on at least days 1 and 8 of the initial treatment course and on at least day 1 of each subsequent treatment course. Contrast-enhanced computed tomography (CT), bone scintigraphy, and diffusion-weighted whole body imaging with background suppression (DWIBS) will be performed at the start of the initial treatment course, after the fifth DTX administration, and at the end of DTX administration. After the end of DTX treatment, clinical examinations will be performed every three months and contrast-enhanced CT, bone scintigraphy, and DWIBS will be performed every six months.

### Data management, control of data consistency, quality control, monitoring, and audits

All study data are collected and managed by the Department of Urology at Kanazawa University. Data consistency and quality are ensured through regular monitoring and internal audits conducted in accordance with institutional regulations.

### Statistical analysis

Approximately 30 patients are planned to be enrolled in this study. The sample size was determined based on feasibility considerations and the exploratory nature of this single-center phase II study. Efficacy and safety analyses are performed using the full analysis set and the per-protocol set. Survival outcomes are estimated using the Kaplan-Meier method.

### Ethics approval

This study was conducted in accordance with the Declaration of Helsinki and relevant ethical guidelines. All treatments and examinations for PC were conducted after written informed consent was obtained before enrollment. This study was approved by the Certified Review Board of Kanazawa University (Approval No. 2024-002).

## Discussion

The TRIC study was designed to address an important unmet clinical need in the management of high-risk mCSPC in Japan. Although triplet therapy combining ADT, DTX, and an ARSI has demonstrated superior survival outcomes in patients with aggressive disease, its real-world applicability is frequently limited by treatment-related toxicity, particularly toxicity associated with DTX ^(10), (11)^. A key strength of the TRIC study is its direct relevance to clinical practice within the Japanese healthcare system. Under the current national health insurance framework, DAR is the only ARSI approved for use in triplet therapy for mCSPC. As a result, treatment optimization must focus on improving the feasibility of DTX-containing regimens rather than selecting alternative ARSIs. In this context, modification of the DTX dosing schedule represents one of the few available strategies to enhance tolerability while maintaining access to treatment intensification. This study is novel in its prospective evaluation of a biweekly low-dose DTX regimen combined with DAR and ADT in the mCSPC setting. Though standard-dose DTX has been extensively studied in combination regimens, limited data are available regarding alternative dosing schedules, particularly in Asian populations, who may be more susceptible to chemotherapy-related toxicities ^(7)^. Our prior experience with biweekly low-dose DTX in mCRPC provides a strong rationale for investigating this approach earlier in the disease course ^(8)^. Although the cumulative dose intensity of the biweekly regimen is lower than that of the conventional 3-weekly schedule, this approach is expected to improve treatment tolerability and feasibility. The TRIC study is designed to prospectively evaluate whether this modified regimen can maintain sufficient clinical activity in this patient population. Another distinctive feature of the TRIC study is the use of the Canazawa risk classification for patient selection. Although the Canazawa risk classification is not widely used internationally, it was developed to capture aggressive disease biology using Gleason pattern 5, BSI, and LDH ^(5)^. Importantly, this classification is not intended as a direct substitute for established criteria such as LATITUDE or Chemohormonal Therapy Versus Androgen Ablation Randomized Trial for Extensive Disease in Prostate Cancer (CHAARTED) and yields a different distribution of risk groups; therefore, the eligibility definition is provided explicitly to facilitate interpretation and generalizability. This risk stratification system incorporates histopathologic features, disease burden quantified by BSI, and biochemical markers, enabling identification of patients with particularly aggressive disease biology ^(5), (6)^. Such a biomarker-informed approach reflects real-world clinical decision-making and may enhance the interpretability and applicability of the study results.

PSA response was selected as the primary endpoint of this exploratory phase II trial to allow early assessment of treatment activity. The threshold of ≥95% PSA decline was selected based on our previous study, in which PSA reduction of <95% at 12 weeks after initiation of ADT was identified as an independent predictor of shorter TTCR across multiple risk classifications ^(5)^. This cutoff was therefore considered a clinically meaningful indicator of deep biochemical response and early treatment activity. PSA decline has not been formally validated as a surrogate endpoint for OS at the trial level, and previous analyses have demonstrated that PSA-based endpoints do not fully capture the survival benefit of systemic therapies in metastatic PC ^(12)^. Nevertheless, a substantial body of evidence indicates that PSA kinetics are strongly associated with clinical outcomes at the individual patient level. Analyses from large cooperative group trials have shown that PSA progression events and early PSA changes are significant predictors of OS in both mCSPC and mCRPC ^(13)^. In addition, measures of PSA kinetics such as PSA velocity and PSA doubling time have been shown to improve survival prediction beyond baseline clinical factors ^(14)^. More recently, post hoc analyses of contemporary phase III trials have demonstrated that early, deep, and durable PSA declines following treatment intensification are associated with favorable long-term outcomes, including improved OS and PFS ^(15), (16)^. These findings suggest that PSA response reflects underlying biological activity and treatment sensitivity, even though it does not meet the strict statistical criteria required for validation as a surrogate endpoint. PSA response represents a pragmatic and informative indicator of early treatment effect in single-arm phase II studies, particularly when the primary objectives include feasibility and preliminary efficacy assessment.

Several limitations of the TRIC study should be acknowledged. First, the single-arm design precludes direct comparison with standard-dose DTX-based triplet therapy or other treatment strategies. Second, the relatively small sample size may limit the generalizability of the findings. Third, the use of PSA response as the primary endpoint does not allow definitive conclusions regarding long-term survival benefit. Accordingly, the results of this study should be interpreted as providing early signals of treatment activity rather than confirmatory evidence of clinical benefit. Longer follow-up and randomized studies will be required to establish the impact of this regimen on survival outcomes.

Despite these limitations, the TRIC study is expected to generate valuable data regarding the feasibility, safety, and early efficacy of a modified triplet therapy tailored to the Japanese clinical setting. The findings of this study will inform the design of future randomized trials and may contribute to the development of optimized treatment strategies for patients with high-risk mCSPC who are candidates for treatment intensification.

## Article Information

### Acknowledgement

Artificial intelligence-based tools (ChatGPT) were used to assist with language editing and sentence refinement during manuscript preparation. The authors take full responsibility for the content and integrity of the manuscript.

### Author Contributions

All authors have reviewed the final version to be published and have agreed to be accountable for all aspects of the work. Concept and design: Hiroaki Iwamoto, Tomohiro Hori, Takahiro Inaba, Ryunosuke Nakagawa, Taiki Kamijima, Hiroshi Kano, Tomoyuki Makino, Renato Naito, Hiroshi Yaegashi, Takahiro Nohara, Kazuyoshi Shigehara, Kouji Izumi, and Atsushi Mizokami. Acquisition, analysis, or interpretation of data: Hiroaki Iwamoto. Drafting of the manuscript: Hiroaki Iwamoto. Critical review of the manuscript for important intellectual content: Hiroaki Iwamoto, Tomohiro Hori, Takahiro Inaba, Ryunosuke Nakagawa, Taiki Kamijima, Hiroshi Kano, Tomoyuki Makino, Renato Naito, Hiroshi Yaegashi, Takahiro Nohara, Kazuyoshi Shigehara, Kouji Izumi, and Atsushi Mizokami.

### Conflicts of Interest

None

### IRB Approval Statement

This study protocol was approved by the Certified Review Board of Kanazawa University (institution ID: CRB4180005; Approval No. 2024-002).

### Statement on Patient Informed Consent

Informed consent is not applicable because this manuscript describes a study protocol and no individual patient data are included.

### Disclosure

Human subjects: Consent for treatment and open access publication was obtained or waived by all participants in this study. TRIC study received approval from the Kanazawa University Clinical Research Review Committee (institution ID: CRB4180005). Animal subjects: All authors have confirmed that this study did not involve animal subjects or tissue.
